# IL-17A^+^GM-CSF^+^ Neutrophils Are the Major Infiltrating Cells in Interstitial Lung Disease in an Autoimmune Arthritis Model

**DOI:** 10.3389/fimmu.2018.01544

**Published:** 2018-07-02

**Authors:** Oh Chan Kwon, Eun-Ju Lee, Eun-Ju Chang, Jeehee Youn, Byeongzu Ghang, Seokchan Hong, Chang-Keun Lee, Bin Yoo, Yong-Gil Kim

**Affiliations:** ^1^Division of Rheumatology, Department of Medicine, College of Medicine, University of Ulsan, Asan Medical Center, Seoul, South Korea; ^2^Department of Biomedical Science, College of Medicine, University of Ulsan, Asan Medical Center, Seoul, South Korea; ^3^Department of Anatomy and Cell Biology, College of Medicine, Hanyang University, Seoul, South Korea

**Keywords:** GM-CSF, IL-17A, neutrophil, autoimmune arthritis, interstitial lung disease

## Abstract

**Objective:**

To gain a better understanding of the pathogenesis of autoimmune arthritis-associated interstitial lung disease (ILD), we sought to identify the characteristics of lung-infiltrating cells in SKG mice with ILD.

**Methods:**

We injected curdlan in SKG mice at 8 weeks of age, and identified the presence of ILD by PET-MRI at 20 weeks post-injection and histological analysis at 22 weeks post-injection. Lung-infiltrating cells were examined by flow cytometry. Analysis of serum cytokines by the Luminex multiplex cytokine assay was performed at 14 and 22 weeks post-injection, and cytokine profiles before and after the development of ILD were compared. Opal multiplexed immunofluorescent staining of lung tissue was also performed.

**Results:**

At 20 weeks post-injection, curdlan-treated SKG mice developed not only arthritis but also lung inflammation combined with fibrosis, which was identified by PET-MRI and histological analysis. The majority of inflammatory cells that accumulated in the lungs of curdlan-treated SKG mice were CD11b^+^Gr1^+^ neutrophils, which co-express IL-17A and GM-CSF, rather than TNF-α. Compared with 14 weeks post-injection, serum levels of GM-CSF, MCP1, IL-17A, IL-23, TSLP, and soluble IL-7Rα had increased at 22 weeks post-injection, whereas those of IFN-γ, IL-22, IL-6, and TNF-α remained unchanged. Furthermore, IL-23, CXCL5, IL-17A, and GM-CSF, but not TNF-α, were observed in immunofluorescent-stained lung tissue.

**Conclusion:**

We found that IL-17A^+^GM-CSF^+^ neutrophils represented the major inflammatory cells in the lungs of curdlan-treated SKG mice. In addition, GM-CSF and IL-17A appear to play a more important role than TNF-α in ILD development.

## Introduction

Interstitial lung disease (ILD) is a common extra-articular manifestation of rheumatoid arthritis (RA) occurring in up to 30% of RA patients (1). Although treatment of RA has markedly improved in recent years with the introduction of biologic therapies, the use of such agents has often been restricted in RA-associated ILD due to safety concerns (2). Following initial reports of a link between anti-TNF therapy and serious respiratory adverse events (SRAEs) (3, 4), subsequent studies have shown an association between all biologic agents used to treat RA and SRAEs (5–8). This discrepancy in the effect of biologic agents on synovial and lung inflammation indicates that the nature of inflammation in the synovium and lung may be different in RA. Thus, a better understanding of the pathogenesis of RA-associated ILD may lead to better treatment approaches.

SKG mice are an animal model in which chronic autoimmune arthritis can be developed (9, 10). These mice possess a mutation in the gene encoding the SH2 domain of ZAP-70, a key signal transduction molecule in T cells (11, 12). This mutation in ZAP-70 results in thymic-positive selection and failure in the negative selection of highly self-reactive T cells that include potentially arthritogenic T cells (9). These self-reactive T cells lead to chronic arthritis and extra-articular manifestation, including ILD (9, 13). Therefore, ILD in SKG mice may be a good candidate for a murine RA-associated ILD model.

In this study, we assessed the characteristics of lung-infiltrating cells in SKG mice with ILD to better understand the pathogenesis of RA-associated ILD. We found that IL-17A^+^GM-CSF^+^ cells increased in lung tissue and were primarily neutrophils. Furthermore, serum levels of IL-17A and GM-CSF increased. These results suggest that IL-17A^+^GM-CSF^+^ neutrophils serve as the major inflammatory cells in this murine RA-associated ILD model.

## Materials and Methods

### Induction of Arthritis and ILD in SKG Mice

Male SKG mice, obtained from Dr. S. Sakaguchi (University of Kyoto, Japan) and male BALB/c were maintained under specific pathogen-free conditions. Disease was induced at 8 weeks of age by administering 3 mg curdlan by intraperitoneal injection; mice were then monitored for up to 22 weeks. All mice were handled in accordance with the guidelines for animal care approved by the Animal Experimentation Committee of the Asan Institute for Life Sciences (2015-14-135).

### PET-MRI Scan

At 20 weeks, whole-body sequential PET/MRI scanning of the mice was performed using a nanoScan PET/MRI (Mediso Ltd.). 18F-FDG (0.2 mCi/kg) was injected into the tail vein after a fasting period of at least 12 h, and a 30-min scan was initiated at 40 min after injection of the radioligand. After the MRI scan for 20 min, PET scan was performed for 10 min. MRI scans were acquired and contiguous axial slices (1 mm) were obtained for the whole body. Scanning parameters were repetition time = 25 s, effective echo time = 3.4 ms, field of view = 64 mm, number of excitations = 1, frequency = 128, and phase = 128. Dynamic data acquisition of PET scans was performed from 60 to 70 min after 18F-FDG injection. Acquired PET images were reconstructed using the 3D full detector mode with MRI-based attenuation collection, with an energy level of 250–750 keV and 0.5-mm voxel size.

### Lung Histology

Mice were euthanized with a mixture of zoletil (Virbac) and Rompun™ (Bayer). The lung was perfused with PBS *via* the heart to remove bronchoalveolar and blood cells. The right lung was inflated with 10% buffered formalin, embedded in paraffin, and sectioned at 4-µm thickness. Sections were then stained with hematoxylin and eosin (H&E) and Masson’s trichrome.

### Surface and Intracellular Staining and Flow Cytometry

Fc receptors were blocked with anti-mouse CD16/32 (BioLegend, clone: 93), and surface markers were stained with BV421-conjugated anti-CD3 (BioLegend, clone: 145-2C11), FITC-conjugated anti-CD4 (BioLegend, clone: RM4-5), Alexa Fluor 647-conjugated anti-CD11b (BioLegend, clone: M1/70), PE-conjugated anti-Gr1 (BioLegend, clone: RB6-8C5), PE-conjugated anti-CD44 (BioLegend, clone: IM7), PerCP/Cy5.5-conjugated anti-CD62L (BioLegend, clone: MEL-14), and APC/Cy7-conjugated anti-CD25 (BioLegend, clone: 3C7). After fixing and perminization, intracellular molecules, including cytokines and transcription factors, were stained with PE/Cy7-IL-17A (BioLegend, clone: TC11-18H10.1), PerCP/Cy5.5-conjugated anti-RORγt (BD, clone: Q31-378), Alexa Fluor 647-conjugated anti-FOXP3 (BioLegend, clone:150D), PerCP/Cy5.5-conjugated anti-GM-CSF (BioLegend, clone: MP1-22E9), and Alexa Fluor 647-conjugated anti-TNF-α (BioLegend, clone: MP6-XT22).

### Analysis of Serum Cytokines by Luminex Multiplex Cytokine Assay

Serum samples were prepared at 14 and 22 weeks post-injection. Blood was allowed to clot for a minimum of 1 h at RT and centrifuged at 16,000 × *g* for 15 min at 4°C. Serum concentrations of the following immune molecules were determined using a magnetic bead-based 10-plex immunoassay: GM-CSF, IFN-γ, IL-6, soluble IL-7Rα (sIL-7Rα), IL-17A (CTLA-8), IL-22, IL-23, MCP-1, TNF-α, and TSLP (customized Procartaplex, Thermo Scientific). Briefly, serum samples were mixed with antibody-linked polystyrene beads on 96-well filter bottom plate and incubated at RT for 2 h on an orbital shaker at 500 rpm. After washing, plates were incubated with biotinylated detection antibody for 30 min at RT. Plates were then washed twice and resuspended in streptavidin-PE. After incubation for 30 min at RT, two additional washes were performed, and the plates were resuspended in reading buffer. Each sample was measured in duplicate along with standards (7-point dilutions) and the buffer control. Plates were read using a Luminex Bio-plex 200 system (Bio-Rad Corp.) for quantitative analysis.

### Immunofluorescent Staining

Using the Opal method (Perkin Elmer), six primary antibodies were sequentially applied to a single slide. Slides were deparaffinized in xylene and rehydrated in ethanol. Antigen retrieval was performed in citrate buffer (pH 6.0) using microwave treatment. Primary rabbit antibodies for CD3 (1:100) were incubated for 1 h in a humidified chamber at RT, followed by detection using the Polymer HRP Ms + Rb. Visualization of CD3 was accomplished using fluorescein opal 520 (1:100), after which the slide was placed in citrate buffer (pH 6.0) and heated using microwave treatment. In a serial fashion, slides were then incubated with primary rabbit antibodies for TNF-α (1:500) for 1 h in a humidified chamber at RT, followed by detection using the Polymer HRP Ms + Rb. TNF-α was visualized using opal 540 (1:100). Slides were again placed in citrate buffer (pH 6.0) and subject to microwave treatment and then incubated with primary rabbit antibodies for IL-23 (1:500) for 1 h in a humidified chamber at RT, followed by detection using the Polymer HRP Ms + Rb. IL-23 was visualized using opal 570 (1:100) and slides were placed in citrate buffer (pH 6.0) for microwave treatment. Slides were then incubated with primary rabbit antibodies for CXCL5 (1:100) for 1 h in a humidified chamber at RT, followed by detection using the Polymer HRP Ms + Rb and visualization using opal 620 (1:100). Slides were again placed in citrate buffer (pH 6.0) and heated using microwave treatment. Slides were then incubated with primary rabbit antibody for IL-17A (1:200) for 1 h in a humidified chamber at RT, followed by detection using the Polymer HRP Ms + Rb. IL-17A and visualization using opal 650 (1:100). Slides were again placed in citrate buffer (pH 6.0) and heated using microwave treatment. Slides were then incubated with the last rabbit antibody for GM-CSF (1:200) for 1 h in a humidified chamber at RT, followed by detection using the Polymer HRP Ms + Rb. GM-CSF was visualized using opal 690 (1:100). Finally, slides were again placed in citrate buffer (pH 6.0) and heated using microwave treatment. Nuclei were subsequently visualized with DAPI (1:500) and the sections were mounted to coverslips using mounting media (Enzo). Stained slides were scanned with a multispectral Vectra scanner and quantitative imaging system (Perkin Elmer). To observe co-localization of neutrophil and IL-17A, slides were incubated with primary rabbit antibody for IL-17A (1:200) and rat antibody for neutrophil (1:50) and secondary antibody, followed by incubation of Opal 540 for IL-17A and Opal 690 for neutrophil. Other procedures were identical as described above.

### Statistical Analysis

All analyses were performed using GraphPad Prism 5 software (GraphPad Software). The Mann–Whitney *U* test was performed for two-group comparison. *P* values of <0.05 were considered statistically significant. Error bars shown in all figures indicate the SEM.

## Results

### Curdlan-Treated SKG Mice Developed ILD

**Figure 1:**
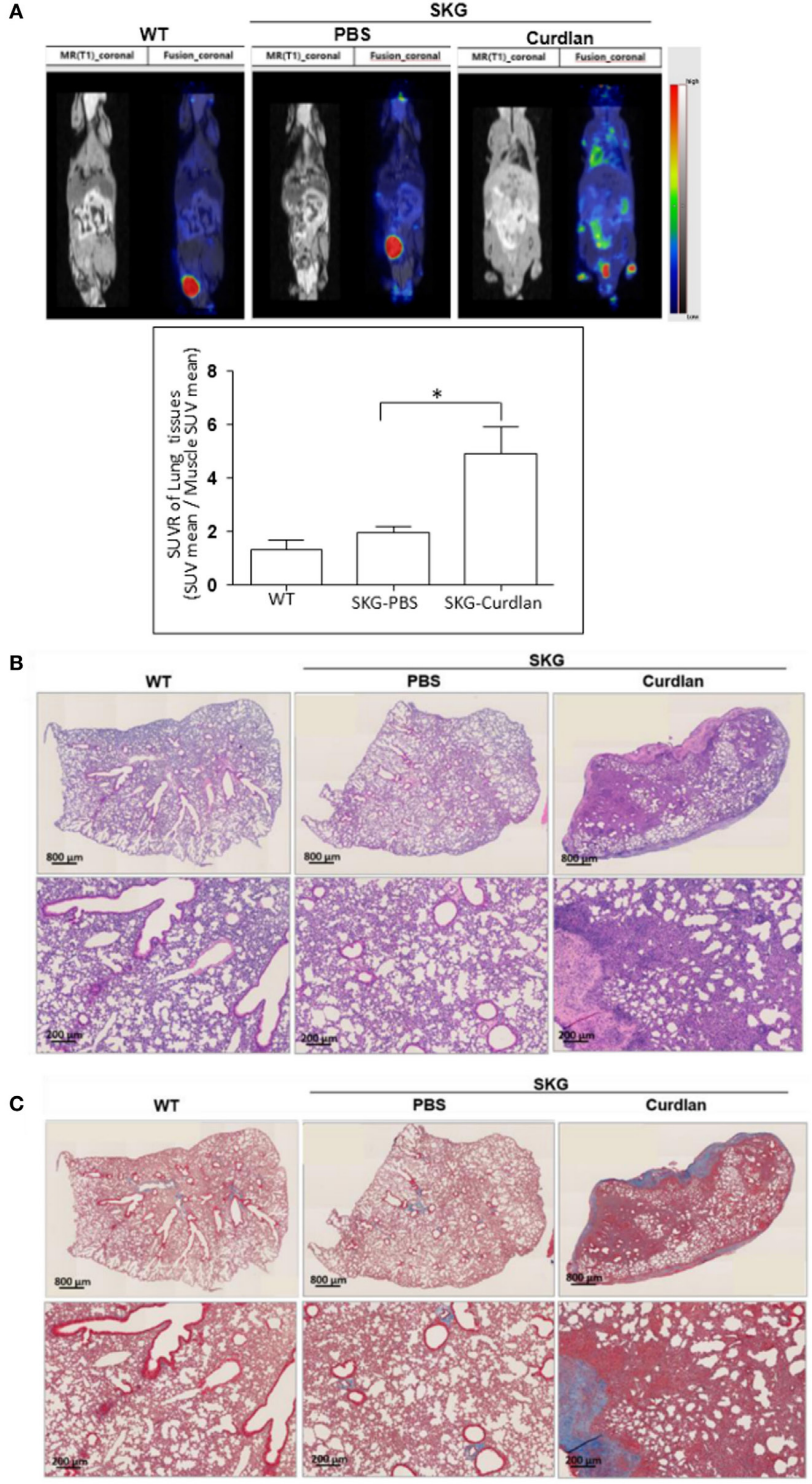
Interstitial lung disease (ILD) in curdlan-treated SKG mice. **(A)** PET-MRI performed at week 20 post-injection revealing inflammation in the peripheral joints, spine, intestine, and lung of the curdlan-treated SKG mice. **(B)** Hematoxylin and eosin staining of the lungs from BALB/c, PBS-treated SKG mice, and curdlan-treated SKG mice at week 22 post-injection showing inflammatory cell infiltration in curdlan-treated SKG mice. **(C)** Masson’s trichrome staining of lungs from BALB/c, PBS-treated SKG mice, and curdlan-treated SKG mice at week 22 post-injection showing fibrosis in the curdlan-treated SKG mice. Values in **(A)** are mean ± SEM. *p < 0.05 by Mann–Whitney *U* test.

From the PET-MRI scans of curdlan-treated SKG mice (*n* = 6), which were taken 20 weeks after curdlan injection, hypermetabolic lesions were observed in the peripheral joints, intestine, and lung (Figure 1A). These hypermetabolic lesions were not detected in BALB/c mice (*n* = 3) or PBS-treated SKG mice (*n* = 5). For the H&E staining of the lung tissue obtained 22 weeks after curdlan injection, the infiltration of inflammatory cells into the interstitial spaces was observed in the curdlan-treated SKG mice (Figure 1B). Masson’s trichrome staining showed that curdlan-treated SKG mice also developed apparent fibrosis (Figure 1C).

### Increased IL-17A^+^GM-CSF^+^ Cells in the Lungs of Curdlan-Treated SKG Mice

**Figure 2:**
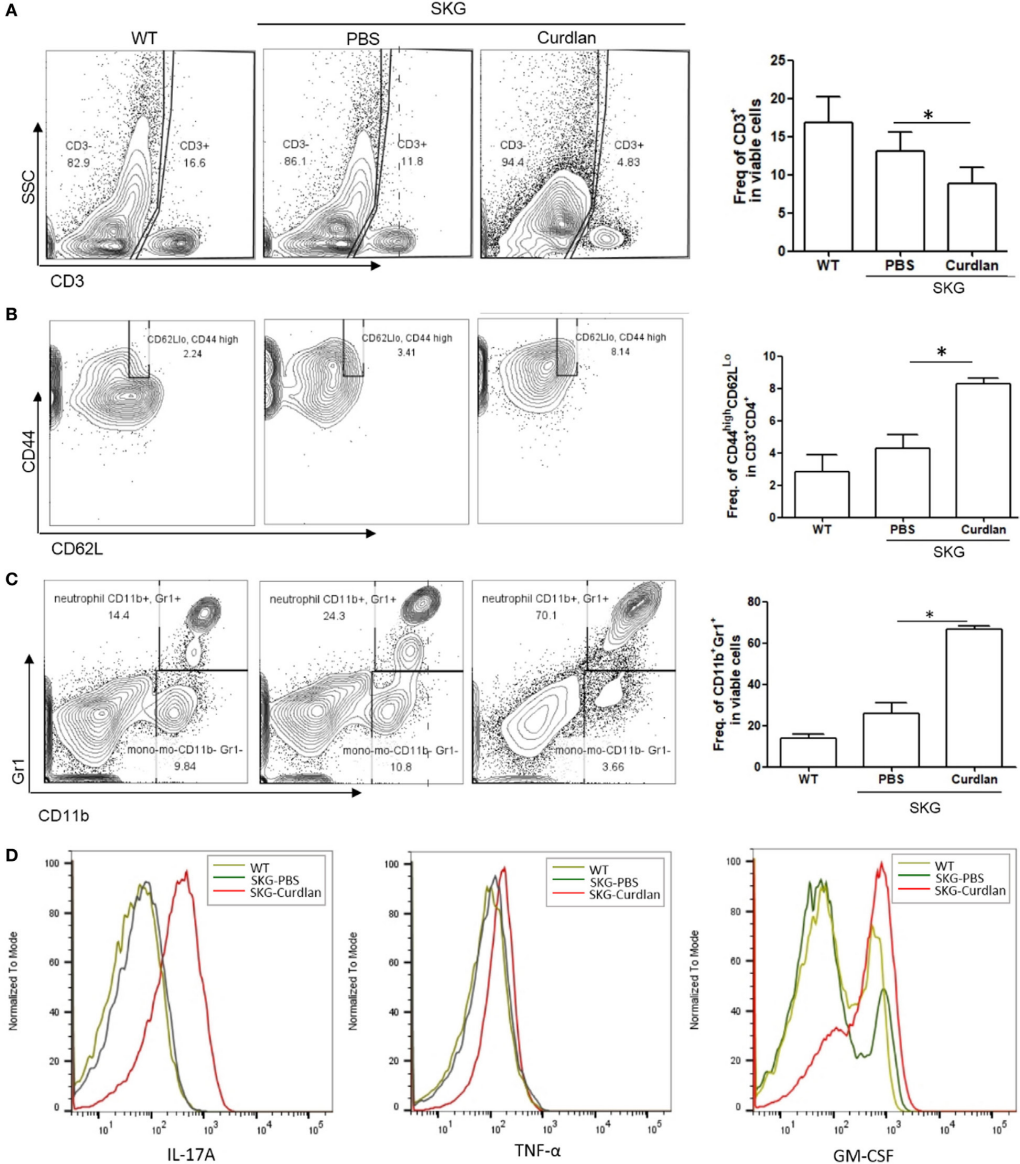
Neutrophils in the lungs of curdlan-treated SKG mice. Flow cytometry plots showing **(A)** proportion of CD3^+^ cells among lung-infiltrating cells, **(B)** proportion of CD44^high^CD62L^Lo^ cells among CD3^+^CD4^+^ cells, and **(C)** proportion of G11b^+^Gr1^+^ cells among lung-infiltrating cells. **(D)** Levels of IL-17A, TNF-α, and GM-CSF in lung-infiltrating cells. Values in **(A–C)** are mean ± SEM. *p < 0.05 by Mann–Whitney *U* test.

We next investigated the characteristics of lung-infiltrating cells in curdlan-treated SKG mice. Flow cytometry plots revealed a lower proportion of CD3^+^ cells (T cells) in the lungs of curdlan-treated SKG mice than in those of BALB/c and PBS-treated SKG mice, although the proportion of effector CD4^+^T cells (CD44^high^CD62L^Lo^ cells) among the CD3^+^CD4^+^ cells was higher (Figures 2A,B). In contrast, proportion of CD11b^+^Gr1^+^ cells (neutrophils) was higher in the lungs of curdlan-treated SKG mice compared with those of BALB/c and PBS-treated SKG mice (Figure 2C). To identify the different features of lung-infiltrating cells among BALB/c, PBS-treated SKG mice, and curdlan-treated SKG mice, we evaluated the expressions of IL-17A, TNF-α, and GM-CSF. Lung-infiltrating cells in the curdlan-treated SKG mice had markedly increased expression of IL-17A and GM-CSF compared with those in BALB/c and PBS-treated SKG mice (Figure 2D).

**Figure 3:**
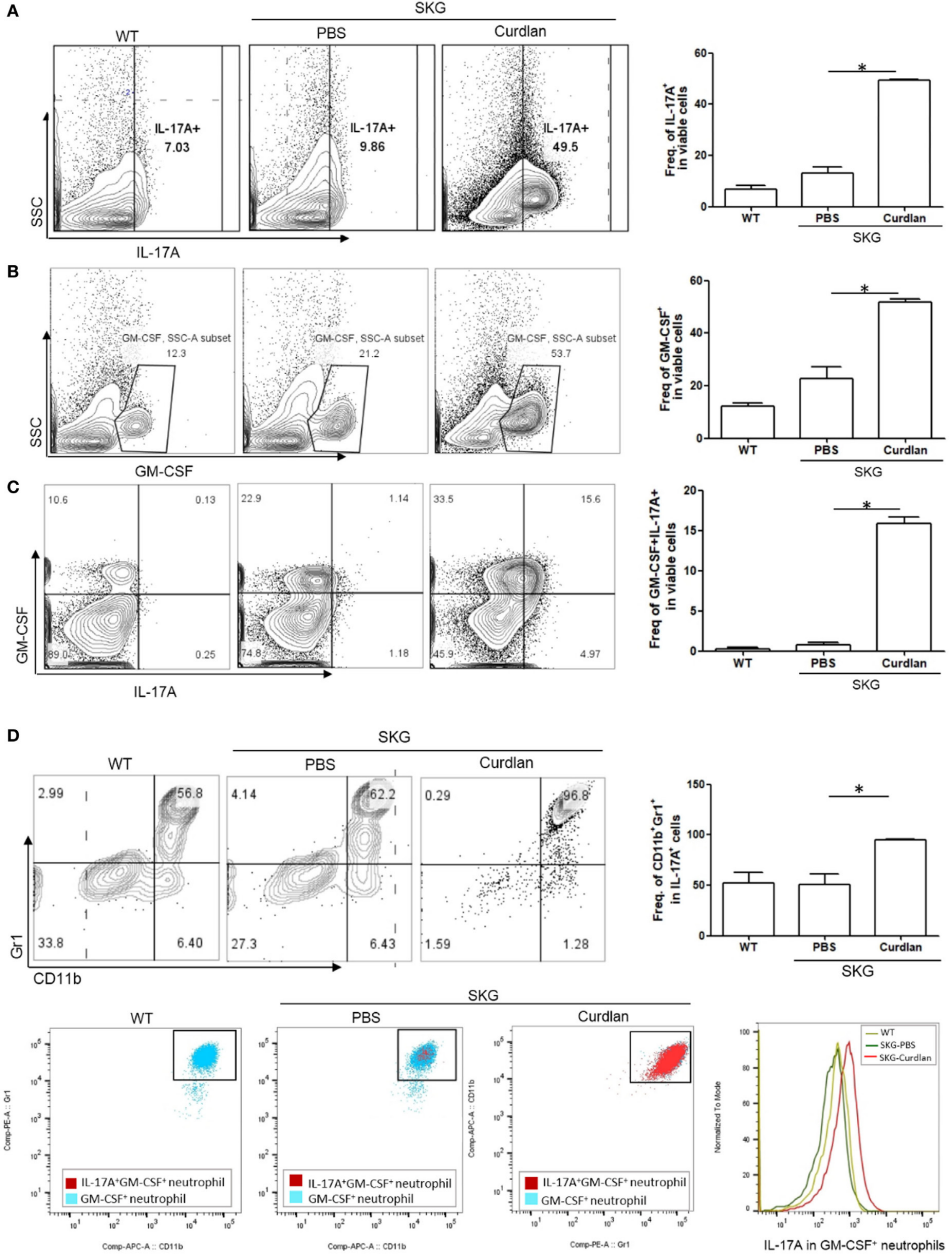
IL-17A^+^GM-CSF^+^ neutrophils in the lungs of curdlan-treated SKG mice. Flow cytometry plots showing **(A)** proportion of IL-17A^+^ cells among lung-infiltrating cells, **(B)** proportion of GM-CSF^+^ cells among lung-infiltrating cells, and **(C)** proportion of IL-17A^+^ and GM-CSF^+^ cells among lung-infiltrating cells. **(D)** Lung-infiltrating cells were gated on IL-17A expression and stained for Gr1 and CD11b. In the lungs of curdlan-treated SKG mice, nearly almost all IL-17A^+^ viable cells were CD11b^+^Gr1^+^ neutrophils, and GM-CSF^+^ neutrophils also expressed IL-17A mostly. Values in **(A–D)** are mean ± SEM. *p < 0.05 by Mann–Whitney *U* test.

In addition, flow cytometry plots showed a significantly higher proportion of IL-17A^+^ and/or GM-CSF^+^ cells in the lungs of curdlan-treated SKG mice compared with those of BALB/c and PBS-treated SKG mice (Figures 3A–C). We further analyzed these IL-17A^+^ cells by gating analysis. In contrast to IL-17A^+^ cells in the lungs of BALB/c and PBS-treated SKG mice, nearly all IL-17A^+^ cells in the curdlan-treated SKG mice were neutrophils (CD11b^+^Gr1^+^). In particular, the majority of GM-CSF^+^ neutrophils in the lungs of curdlan-treated SKG mice expressed IL-17A (IL-17A^+^GM-CSF^+^ neutrophils) (Figure 3D).

### Increased Inflammatory Indices in the Serum of Curdlan-Treated SKG Mice With ILD

**Figure 4:**
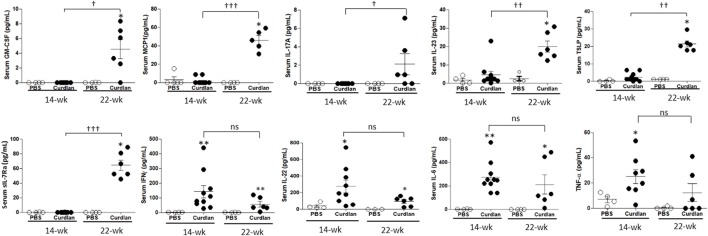
Levels of serum cytokines at week 14 and 22 post-injection in PBS- and curdlan-treated SKG mice. Values are mean ± SEM. *p < 0.05 and **p < 0.01 between PBS- and curdlan-treated mice; ^†^*p* < 0.05; and ^††^*p* < 0.01; ^†††^*p* < 0.001 between 14- and 22-week post-injection in curdlan-treated mice by Mann–Whitney *U* test.

We next measured serum cytokine levels. Compared with curdlan-treated SKG mice at 14 weeks post-injection when peripheral arthritis, but not lung inflammation (Figure S1 in Supplementary Material), was evident, curdlan-treated SKG mice at 22 weeks post-injection (when lung inflammation was also evident) had higher serum levels of GM-CSF, MCP1, IL-17A, IL-23, TSLP, and sIL-7Rα. However, the serum levels of IFN-γ, IL-22, IL-6, and TNF-α did not differ significantly between the two time-points. However, at 14 weeks post-injection, curdlan-treated SKG mice had higher serum levels of IFN-γ, IL-22, IL-6, and TNF-α compared with PBS-treated SKG mice. On the contrary, levels of these markers seemed to decrease at 22 weeks post-injection without statistical significance (Figure 4).

### High Expression of IL-23, CXCL5, IL-17A, and GM-CSF in the Lungs of Curdlan-Treated SKG Mice

**Figure 5:**
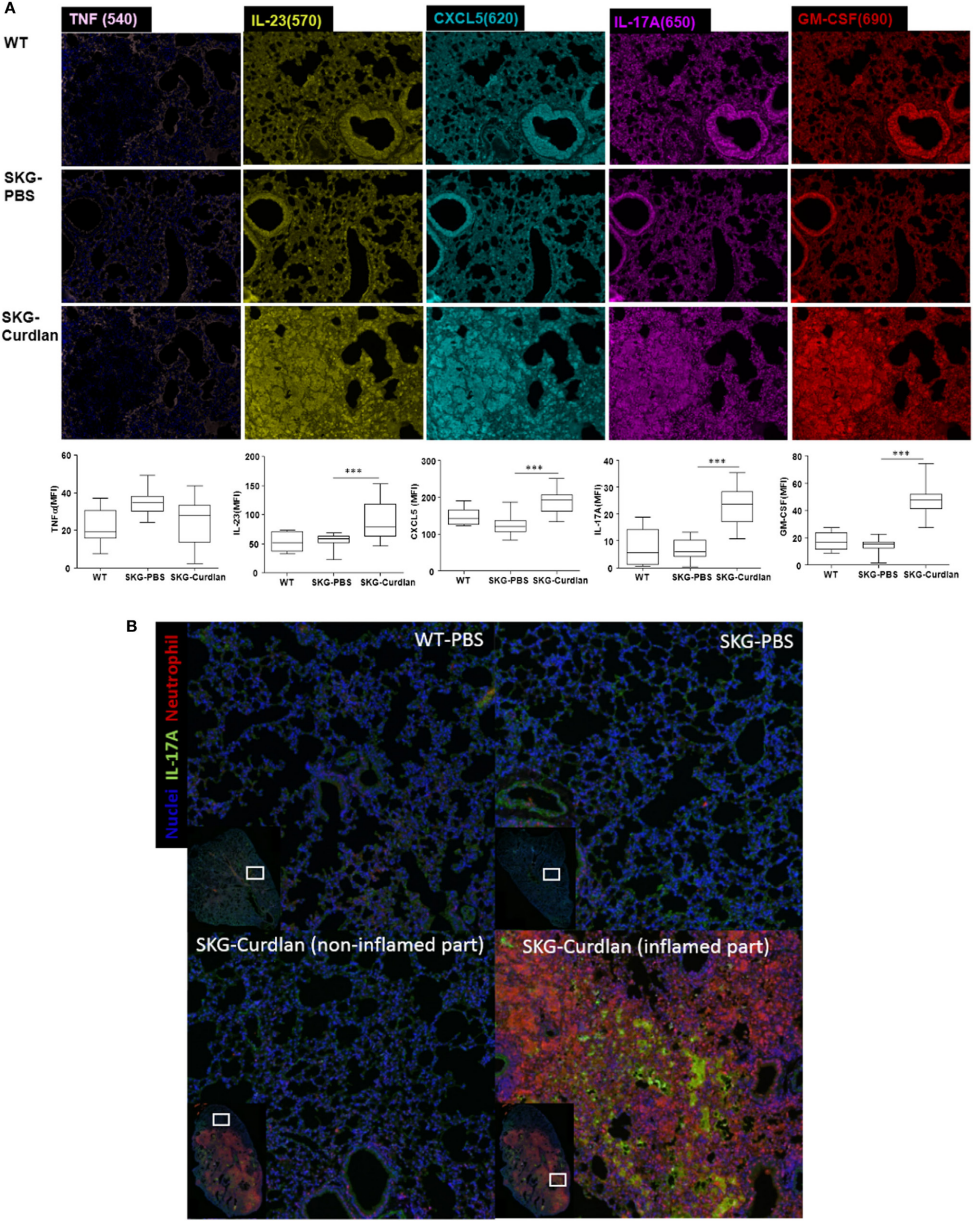
Opal multiplexed immunofluorescent images of lung tissues. **(A)** IL-23^+^, CXCL5^+^, IL-17A^+^, GM-CSF^+^, and TNF-α^+^ cells in curdlan-treated SKG mice. **(B)** IL-17A^+^ cells and neutrophils in inflamed lesion of the lungs of curdlan-treated SKG mice.

We next investigated the lung tissue using Opal multiplexed immunofluorescent staining. The densities of IL-23^+^, CXCL5^+^, IL-17A^+^, and GM-CSF^+^ cells were higher in the curdlan-treated SKG mice than in BALB/c and PBS-treated SKG mice, whereas the density of TNF-α^+^ cells was not different (Figure 5A). This finding was similar to the serum cytokine levels at 22 weeks post-injection in curdlan-treated SKG mice in which IL-23, IL-17A, and GM-CSF increased but not TNF-α (Figure 4). Furthermore, high density of IL-17A^+^ cells and neutrophils were co-localized in inflamed lesions of the lungs of curdlan-treated SKG mice (Figure 5B), thereby demonstrating infiltration of IL-17A^+^ neutrophils in lungs of curdlan-treated SKG mice.

## Discussion

In this study, we found that IL-17A^+^GM-CSF^+^ neutrophils increased in the inflamed lung tissue of curdlan-treated SKG mice. In addition, neutrophils, rather than T cells, were the major inflammatory cells in the lungs of curdlan-treated SKG mice. A previous study evaluating the role of T cells in SKG mice (14) showed that T cells are important mediators of interstitial pneumonitis. In that study, self-reactive T cells from the arthritic joints of SKG mice were capable of mediating not only arthritis but also interstitial pneumonitis. This indicates that inflammation in the lung may be mediated, at least in part, by self-reactive T cells in SKG mice. However, the inflammatory process became less T cell-dependent in the later phase of lung inflammation in that study, implicating that other cells are also involved in lung inflammation. Based on our results, neutrophils seem to play a more important role than T cells. In fact, analyses of inflammatory cells using bronchoalveolar lavage of CTD-associated ILD patients has shown an accumulation of neutrophils, with or without an increased percentage of lymphocytes (15), and neutrophils have been reported as important effector cells associated with poor outcome in CTD-associated ILD (15–20). Concordant with these findings, our results also showed that neutrophils, particularly IL-17A^+^GM-CSF^+^ neutrophils, as seen by flow cytometry of the lungs of curdlan-treated SKG mice, are the most important inflammatory cells in ILD.

Recently, Sendo et al. (21) analyzed lung-infiltrating cells in zymosan A-treated SKG mice. They reported that CD11b^+^Gr1^+^, Th17, and innate lymphoid cells increased in the lungs of zymosan A-treated SKG mice, with varying proportions of these cells according to ILD severity. Our results provide additional information by showing that the CD11b^+^Gr1^+^ cells in the lungs of SKG mice with ILD co-express IL-17A and GM-CSF. Sendo et al. (21) also reported a new cell population, CD11b^+^Gr1^dim^ tolerogenic dendritic cell (DC)-like cells, which appeared to suppress the development of ILD in SKG mice and was increased only in the severely inflamed lung tissue (defined by ≥60% affected). In the present study, this CD11b^+^Gr1^dim^ tolerogenic DC-like cell population was less observed in the flow cytometry plots of lung-infiltrating cells (Figure 2C). Compared with the previous study, in our model, lung inflammation was less severe (<60% affected), whereas lung fibrosis was more severe, resembling the late stage of ILD. The absence of CD11b^+^Gr1^dim^ tolerogenic DC-like cells in our study, in conjunction with more prominent fibrosis as seen by histology, suggests that CD11b^+^Gr1^dim^ tolerogenic DC-like cells may not be observed in the late stage of ILD. Although it remains unclear, further studies elucidating the pathogenic link between these CD11b^+^Gr1^dim^ tolerogenic DC-like cells and IL-17A^+^GM-CSF^+^CD11b^+^Gr1^+^ cells observed in our study may lead to a better understanding of the pathogenic mechanisms of ILD in SKG mice.

In this study, we found that curdlan-treated SKG mice at 22 weeks post-injection had significantly elevated serum levels of GM-CSF, MCP1, IL-17A, IL-23, TSLP, and sIL-7Rα compared with samples taken at 14 weeks post-injection. We speculate that these cytokines are particularly associated with lung inflammation and fibrosis, rather than arthritis, since they were increased only at 22 weeks post-injection. There is a body of evidence supporting GM-CSF as an important mediator in lung inflammation by upregulating TLR2, TLR4, and CD14 expression (22–24), and by boosting IL-6 and IL-1β production from macrophages (22, 25, 26). In addition, GM-CSF itself is a strong inducer of neutrophil infiltration in various tissues (27, 28). Furthermore, the neutralization of GM-CSF has been shown to significantly inhibit neutrophil infiltration into the lungs of SKG mice, showing the importance of GM-CSF in lung inflammation (29).

Although a previous study showed that blocking IL-17A was not effective in preventing ILD development (29), IL-17A is also known as a strong inducer of neutrophil infiltration (30). In our data, the lung-infiltrating cells were mostly CD11b^+^Gr1^+^ neutrophils and these cells co-express GM-CSF as well as IL-17A. A previous study showed that gamma/delta T cells are the predominant source of IL-17 in mice with collagen-induced arthritis, but not in SKG mice (31). Our results suggest that neutrophils might be the main source of IL-17 in curdlan-treated SKG mice, especially when both arthritis and ILD are present. In these circumstances, where neutrophils play a major role in ILD pathogenesis such as in our study, blocking IL-17A may have a role in controlling ILD aggravation. Recent report by Miyachi et al. (32) showed improvement of ILD in patients with psoriasis through the use of secukinumab (anti-IL-17A monoclonal antibody), thus supporting IL-17A blockade as a potential treatment option for ILD.

MCP1, which is known as a monocyte chemoattractant, is also important in the recruitment of neutrophils to the lung (33). Thus, increased serum levels of MCP1 might have played a role in increased neutrophil infiltration in mouse lungs in our study. In a recent study, IL-23-treated neutrophils produced IL-17A selectively, and IL-17^+^ neutrophils were found in the colons of a DSS-induced colitis model through the adoptive transfer of IL-23-treated neutrophils (34). Moreover, IL-23-mediated pathways are known to drive inflammation in various tissues including intestine in curdlan-treated SKG mice (35); IL-23 also triggers production of GM-CSF in the lung (36), which may lead to GM-CSF^+^ polarization of neutrophils in the lung. Taken together, increased serum levels of IL-23 in our study may have contributed to IL-17A^+^GM-CSF^+^ polarization of neutrophils in the lungs of SKG mice.

TSLP, which was also increased in the serum of curdlan-treated SKG mice at 22 weeks post-injection, was first implicated as a driver of Th2 responses in the airway (37). Aberrant levels of TSLP have been observed in a variety of airway diseases, such as asthma, chronic obstructive pulmonary disease, and nasal polyps (38, 39). Recently, TSLP has also emerged as an important cytokine in the pathogenesis of systemic sclerosis and idiopathic pulmonary fibrosis (40–42). The biologic effects of TSLP are mediated by binding to a functional heterodimeric receptor complex composed of the TSLP receptor and the sIL-7Rα chain (43), which signals through the STAT3 (44) and STAT5 pathways (45). The elevated serum levels of TSLP and sIL-7Rα observed in our study may account for the fibrosis of the lung tissue, although the mechanisms by which TSLP and sIL-7Rα are increased have yet to be elucidated.

We speculate that increased serum levels of GM-CSF, MCP1, and IL-17A in curdlan-treated SKG mice collectively promote neutrophil recruitment into the lung. The neutrophils are then polarized to IL-17A^+^GM-CSF^+^ neutrophils, probably by increased serum level and tissue expression of IL-23. Moreover, the increased expression of IL-17A and GM-CSF within the lung tissues further recruits more neutrophils into the lung, thereby contributing to a positive feedback loop of neutrophil infiltration and polarization. This can be a possible explanation for why IL-17A^+^GM-CSF^+^ neutrophils are the major infiltrating cells in ILD of curdlan-treated SKG mice. It is also possible that the increased serum levels of TSLP and sIL-7Rα contributed to lung fibrosis.

A previous study suggested IL-6, TNF-α, and IL-1 as key cytokines that mediate autoimmune arthritis in SKG mice (46). Consistently, our data also showed that at 14 weeks post-injection, curdlan-treated SKG mice had higher serum levels of IL-6 and TNF-α compared with PBS-treated SKG mice. Furthermore, serum levels of IL-6 and TNF-α seemed to be numerically lower in the curdlan-treated SKG mice at 22 weeks post-injection than that at 14 weeks post-injection. This implies that IL-6 and TNF-α may not be as important in lung inflammation as it is in arthritis. This finding may account for the observation that anti-TNF and anti-IL-6R agents are useful for treating arthritis, but not for ILD. Indeed, in the Opal staining, the population of TNF-α^+^ cells was not increased. Rather, populations of IL-23^+^, CXCL5^+^, IL-17A^+^, and GM-CSF^+^ cells were increased. Thus, these cytokines may play a role in developing RA-associated ILD.

In summary, we observed lung inflammation and fibrosis mimicking ILD, following autoimmune arthritis, and identified IL-17A^+^GM-CSF^+^ neutrophils as the main inflammatory cell population in the inflamed lung tissue of curdlan-treated SKG mice. We also demonstrated GM-CSF and IL-17A, all of which were increased in the serum at the time of ILD development, suggesting IL-17A^+^GM-CSF^+^ neutrophils are a major mediator of pathogenesis in this ILD model. Further elucidation of the exact mechanisms by which IL-17A^+^GM-CSF^+^ neutrophils are induced, and how they mediate lung inflammation and fibrosis, may lead to a better understanding of the pathogenic mechanisms of RA-associated ILD.

## Ethics Statement

This study was carried out in accordance with the guidelines for animal care approved by the Animal Experimentation Committee of the Asan Institute for Life Sciences (2015-14-135).

## Author Contributions

Y-GK designed the study. E-JL performed the experiments. OK, E-JL, E-JC, BG, SH, C-KL, BY, and Y-GK analyzed the data. JY provided the SKG mice. OK, E-JL, and Y-GK wrote the manuscript.

## Conflict of Interest Statement

The authors declare that the research was conducted in the absence of any commercial or financial relationships that could be construed as a potential conflict of interest.

## Supplementary Material

Figure S1 PET-MRI performed in curdlan-treated SKG mice at week 14 post-injection.
