# Supplementary figures and images for: IL-17A+GM-CSF+ Neutrophils Are the Major Infiltrating Cells in Interstitial Lung Disease in an Autoimmune Arthritis Model
Source: Front Immunol. 2018 Jul 2;9:1544. doi: 10.3389/fimmu.2018.01544 (PMC6036238; doi:10.3389/fimmu.2018.01544)

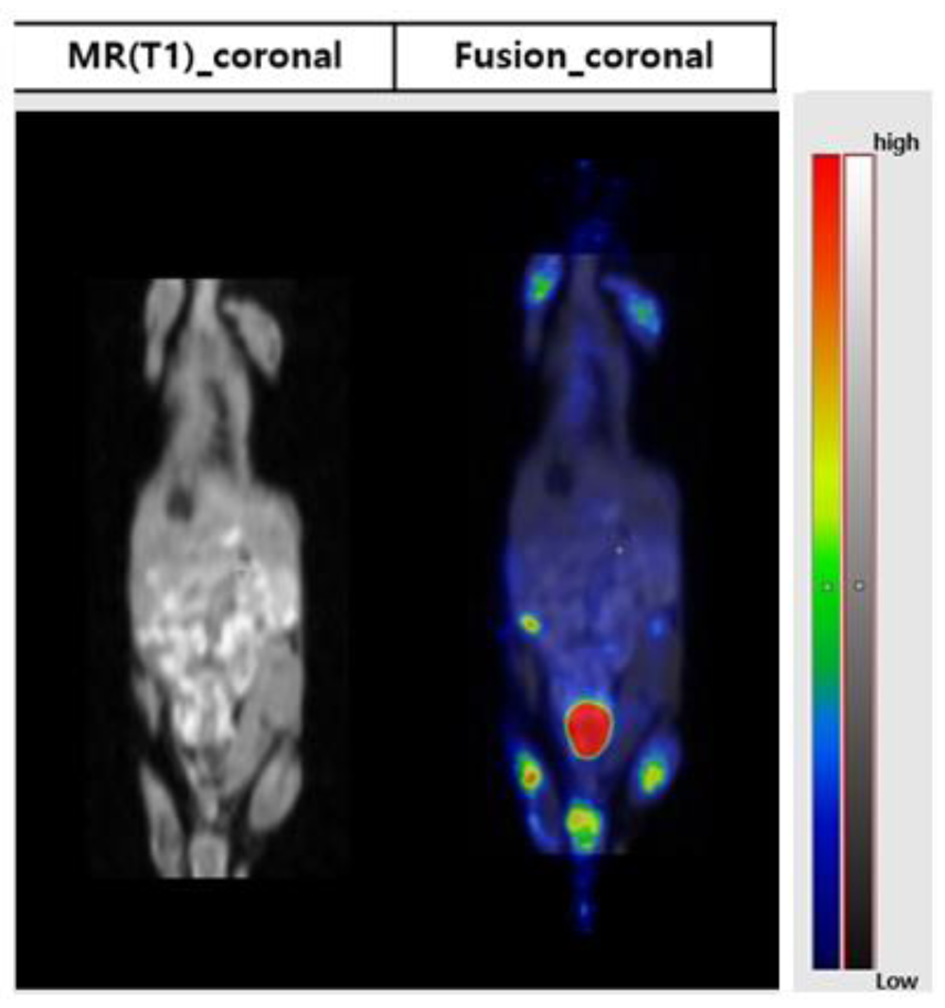

Supplement: Figure S1 — PET-MRI performed in curdlan-treated SKG mice at week 14 post-injection. [file image_1.TIFF]
